# Attitudes, Anxiety, and Behavioral Practices Regarding COVID-19 among University Students in Jordan: A Cross-Sectional Study

**DOI:** 10.4269/ajtmh.20-0418

**Published:** 2020-07-08

**Authors:** Amin N. Olaimat, Iman Aolymat, Nour Elsahoryi, Hafiz M. Shahbaz, Richard A. Holley

**Affiliations:** 1 Department of Clinical Nutrition and Dietetics, Faculty of Applied Medical Sciences, The Hashemite University, Zarqa, Jordan;; 2 Department of Cellular and Molecular Physiology, Faculty of Health and Life Sciences, School of Medicine, University of Liverpool, Liverpool, United Kingdom;; 3 Department of Basic Medical Sciences, Faculty of Medicine, The Hashemite University, Zarqa, Jordan;; 4 Department of Nutrition, Faculty of Pharmacy and Medical Sciences, University of Petra, Amman, Jordan;; 5 Department of Food Science and Human Nutrition, University of Veterinary and Animal Sciences, Lahore, Pakistan;; 6 Department of Food and Human Nutritional Sciences, University of Manitoba, Winnipeg, Canada

## Abstract

The COVID-19 pandemic represents a major public health, economic, political, and scientific concern in most countries around the globe where COVID-19 cases and deaths have been confirmed. This study assessed the attitudes, anxiety, and behavioral practices of university students in Jordan regarding COVID-19 during the early period of the infection (March 19–21, 2020) using a validated, self-administered survey questionnaire. Positive attitudes or low-risk practices were given 1 point, whereas negative attitudes or high-risk practices were scored 0. Percentages of the total score were used for categorizing data into negative attitudes or high-risk practices (≤ 60%), moderate attitudes or moderate-risk practices (60.01–80%), and positive attitudes or low-risk practices (> 80%). Generally, the university students displayed positive attitudes and low-risk practices toward preventing COVID-19, with an average score of 81.1% and 84.3%, respectively. Approximately two-thirds (69.1%) of the students showed a positive attitude toward COVID-19 seriousness, concern of contracting the virus, and the appropriate prevention measures, and low-risk practices (67.6%) toward preventing COVID-19 including implementation of social distancing and good hygiene. Female, older, medical, or postgraduate students practiced significantly more (*P* ≤ 0.05) appropriate hygiene and social distancing behaviors toward COVID-19 than their counterparts of each group. More than two-thirds (69.2%) of the students were anxious that they might become infected with COVID-19. These results are important for health authorities to develop appropriate educational programs and protective health measures including good respiratory etiquette and handwashing practices, to enhance safer lifestyles and prevent COVID-19 transmission.

## INTRODUCTION

In December 2019, a human respiratory disease initially appeared in Wuhan, China, caused by a novel member of the coronaviruses, characterized as SARS-CoV-2, and called COVID-19.^1–3^ It has been suggested that COVID-19 was originally endemic in bats, and then transmitted to humans via wild animals; however, the subsequent rapid spread of the virus was through human-to-human transmission.^4^ Most of the identified coronaviruses cause mild human disease, except for the highly pathogenic, SARS-CoV and Middle East respiratory syndrome coronavirus (MERS-CoV), which are associated with complicated infections and fatalities.^5^ COVID-19 is more contagious than MERS-CoV and SARS-CoV, and, not surprisingly, the WHO declared that the spread of COVID-19 had become a pandemic on March 11, 2020.^6^ The WHO confirmed that there have been at least 8 million COVID-19 cases and almost 435,000 deaths worldwide.^6^ The Jordanian Ministry of Health has reported 987 confirmed COVID-19 cases and nine deaths in Jordan till June 17, 2020.^7^

In most of the infected cases, the illness is self-limiting, and patients recover completely.^3,8^ The virus may cause severe infections resulting in septic shock, acute respiratory distress syndrome, acute cardiac or kidney injury, multi-organ failure, and death.^1–4,9^ Adults and children usually develop mild self-limiting disease, whereas more severe COVID-19 cases were observed in the elderly or in people of all ages with underlying medical conditions such as cardiovascular diseases, obesity, cancer, and diabetes.^3,8^

COVID-19 is mainly transmitted from person to person via sneezing or cough-induced respiratory droplets from the mouth or nose of infected persons.^3,6,10^ Other modes of COVID-19 transmission might involve touching surfaces contaminated with the virus followed by touching the mouth or the eyes.^6,11^ A history of contact with infected patients in the past 2 weeks should be taken as a reason to self-isolate to reduce further spread of COVID-19 infection.^4^

To date, there is no vaccine or curative therapy available for COVID-19. Most of the current treatments used are only able to alleviate the symptoms, and severe illness is supported by mechanical ventilation. Although some antiviral, anti-inflammatory, and antimalarial drugs have been used to treat COVID-19, none of these medications has been approved as an effective therapeutic solution for COVID-19.^10,12^

Therefore, so far prevention is the only approach available to us to control COVID-19 transmission via providing the public with comprehensive information and good knowledge about the disease to foster positive attitudes and beliefs that enable practicing self-quarantine, social distancing, and good hygienic measures such as handwashing, eye protection, and use of face masks and disposable gloves. Therefore, the knowledge, attitudes, and practices followed by the public play an important role in the planning and enhancement of health initiatives necessary to control the COVID-19 pandemic.

In Jordan, the frequency of COVID-19 cases is increasing progressively but slowly. Similar to many other countries, the government of Jordan adopted preventative measures on March 18, 2020 to control COVID-19 dissemination including border closures, suspending the operation of all government and private institutions except vital sectors, implementing a nationwide curfew, suspension of public transportation, and isolation of infected patients and their close contacts. In addition to these measures, the awareness, attitudes, and practices of people toward COVID-19 could be keys for controlling the spread of the infection. Furthermore, the anxiety and fear due to the presence of pandemic situations may influence the behaviors of individuals. Anxiety provoked in individuals about becoming infected with COVID-19, given the evident global effects, may prompt them to adopt preventative behaviors.^13,14^ The aim of the current study was to assess the attitudes, anxiety and behavioral practices toward COVID-19 among university students in Jordan.

## MATERIALS AND METHODS

### Sample size, study design, and participants.

The sample size was calculated based on a 50% response rate, a 99% CI, and a 5% margin of error with a total student population of 377,000 enrolled in government and private Jordanian universities. The required sample size was 663, and, in this study, the sample size used was 2,083 students, which is 3-fold larger than that required. This cross-sectional study was conducted on 19–21 of March, a few days after COVID-19 detection in Jordan. An online, self-administered questionnaire was used to collect the data. Initially, the questionnaire was written in English, and the content was validated by a group of technical experts in the fields of community health and microbiology to give their professional judgment about clarity and understanding of questions and evaluation of the survey structure. Subsequently, the questionnaire was translated into Arabic. The Arabic version of the questionnaire was validated by a professional translator. The questionnaire was tested for overall acceptability and reliability by conducting a pilot study with 30 respondents who were not included in the formal investigation, and further amendments were carried out. The internal consistency of the attitudes and practices questionnaire was acceptable (Cronbach’s alpha > 0.70). The questionnaire was confirmed after discussions with technical experts. Because of the complete lockdown in Jordan, the data were collected via an online method using Google form. The questionnaire and the positive attitudes and low-risk practices were designed and developed based on the data available on the websites of the WHO,^6^ the CDC,^15^ and the European CDC (ECDC).^16^

The survey link was posted on the student-online-teaching groups such as those on WhatsApp and Facebook which were initiated during the quarantine. The questionnaire included a short introduction to the COVID-19 pandemic, and then the objectives and information on voluntary participation and the anonymity and confidentiality of participation were given. The questionnaire was divided into five parts: the first part requested information on sociodemographic characteristics such as age, gender, university location, level of tertiary study, college, place of residence, and the type of accommodation. The second part of the questionnaire included knowledge assessment and source of COVID-19 information questions, which was published elsewhere.^17^ Attitudes toward COVID-19 seriousness, concern of contracting the virus, and the appropriate prevention measures were explored with six yes/no questions which were assessed in the next part of the questionnaire. In the fourth part, practices of participants toward preventing COVID-19 including implementation of social distancing and good hygiene practices were assessed using 20 yes/no questions. The last section of the questionnaire included two yes/no-based questions to assess the students’ anxiety of being infected with COVID-19 and the effect of the COVID-19 pandemic on the students’ daily life.

### Ethics approval.

This study has been reviewed and approved (May 4, 2019/2020) by the Institutional Review Board Committee of the Hashemite University. Furthermore, an informed consent form showing that participation in this study was fully voluntary and that the participants could withdraw from the survey questionnaire at any point was published on the first page of the online questionnaire. The form was required from all participants prior their participation in the survey.

### Statistical analysis.

The collected data were entered and coded in Microsoft Excel (Microsoft Corporation, Redmond, WA). Simple descriptive statistics were produced for all variables. A scoring system was performed to assess the level of the students’ attitude and behavioral practice toward COVID-19. Each answer reflecting a positive attitude or low-risk practice was given 1 point, whereas a negative attitude or high-risk practice was scored 0. The total attitude or practice score was calculated and converted to percentages. Subsequently, attitudes or practices were then categorized as follows: negative attitude or high-risk practice (≤ 60% of the total score), moderate attitude or moderate-risk practice (60.01–80% of the total score), and positive attitude or low-risk practice (> 80% of the total score).

Categorical variables were analyzed using Pearson’s chi square (χ2) test to investigate the difference between sociodemographic characteristics of the students. *P* ≤ 0.05 was considered statistically significant. All data were analyzed by using the Statistical Package for Social Sciences (SPSS) version 25 (IBM Corporation, Armonk, NY).

## RESULTS

### Demographic characteristics of participants.

Among 2,083 participants, 75.5% were female and 24.5% were male. According to the level of tertiary study, 90.2% and 9.8% of participants were undergraduate or postgraduate students, respectively. The percentages of participants according to fields of study were from human sciences (36.3%), medical sciences (25.7%), engineering sciences (19.9%), and agriculture and general sciences (18.1%). The majority of students (62.6%) were within the ages of 20–24.9 years. The detailed demographic characteristics of participating students, including place of residence, university location, and type of accommodation are presented in Table 1.

**Table 1:** Attitude categories and scores of university students in Jordan toward COVID-19

Demographic variable	Category	*N*	Attitude category, *N* (%)			Attitude mean (%)	SD	χ2	*P*-value
			Negative	Moderate	Positive				
Overall	–	2,083	135 (6.5)	509 (24.4)	1,439 (69.1)	81.1	14.7	–	–
Age (years)	18–19.9	498	34 (6.8)	131 (26.3)	333 (66.9)	80.7	14.9	2.394	0.664
	20–24.9	1,304	82 (6.3)	317 (24.3)	905 (69.4)	81.1	14.6		
	≥ 25	281	19 (6.8)	61 (21.7)	201 (71.5)	81.8	14.6		
Gender	Female	1,572	105 (6.7)	397 (25.3)	1,070 (68.1)	80.8	14.7	3.105	0.212
	Male	511	30 (5.9)	112 (21.9)	369 (72.2)	81.9	14.6		
University location	Northern	551	38 (6.9)	120 (21.8)	393 (71.3)	81.6	14.6	3.637	0.457
	Middle	1,477	95 (6.4)	374 (25.3)	1,008 (68.2)	80.9	14.8		
	Southern	55	2 (3.6)	15 (27.3)	38 (69.1)	80.6	13.9		
College	Engineering	415	29 (7.0)	97 (23.4)	289 (69.6)	81.2	15.0	6.388	0.381
	Medical sciences	535	40 (7.5)	119 (22.2)	376 (70.3)	81.2	14.8		
	Agriculture and general sciences	376	17 (4.5)	104 (27.7)	255 (67.8)	81.5	14.3		
	Human sciences	757	49 (6.5)	189 (25)	519 (68.6)	80.7	14.6		
Level of tertiary study	Undergraduate	1,879	120 (6.4)	462 (24.6)	1,297 (69.0)	81.1	14.7	0.455	0.797
	Postgraduate	204	15 (7.4)	47 (23.0)	142 (69.6)	80.8	14.5		
Accommodation type	Villa	106	5 (4.7)	29 (27.4)	72 (67.9)	80.5	15.0	4.878	0.560
	Flat	798	48 (6)	197 (24.7)	553 (69.3)	81.1	14.3		
	House	1,153	80 (6.9)	273 (23.7)	800 (69.4)	81.2	14.9		
	Others	26	2 (7.7)	10 (38.5)	14 (53.8)	79.5	18.4		
Place of residence	City	1,642	111 (6.8)	413 (25.2)	1,118 (68.1)	80.7	14.7	4.545	0.337
	Village	390	22 (5.6)	87 (22.3)	281 (72.1)	82.2	14.8		
	Others	51	2 (3.9)	9 (17.6)	40 (78.4)	85.3	14.0		

### Attitudes of students toward COVID-19.

Regardless of the demographic variables, students who participated in this study displayed positive attitudes toward COVID-19 with an average score of 81.1%, which is considered to be good. About two-thirds of the students (69.1%) showed a good attitude, whereas a quarter (24.4%) of them showed what was considered a moderate attitude. On the other hand, a small proportion of the students (6.5%) exhibited a negative attitude. All tested sociodemographic variables had no significant association (*P* > 0.05) with the attitude of the students toward COVID-19. However, positive attitude scores and age were somewhat correlated, with about 71.5% of students aged ≥ 25 years showing a positive attitude score toward COVID-19 compared with 66.9% of students aged < 20 years. Furthermore, the proportion of male students who showed a positive attitude toward COVID-19 was larger than that of female students. Medical students had the highest attitude score toward COVID-19 among students from all other study fields surveyed (Table 1).

Among six attitude questions toward COVID-19, more than 80% of students correctly answered four questions. The majority of students (≥ 98%) believed that COVID-19 could be controlled by applying isolation measures for infected people or by the awareness of the population about the disease. Furthermore, 96.3% of the students were interested in knowing the methods for COVID-19 prevention. More than four-fifths (81.5%) of the students believed that COVID-19 is a dangerous disease. Nonetheless, only 63.6% of the students considered that they were vulnerable to COVID-19 infection, and about one-half (47.8%) of the students considered that the available information about COVID-19 was sufficient (Table 2).

**Table 2:** Responses about attitudes toward COVID-19 among university students in Jordan

Question (positive attitude)	Positive attitude, *N* (%)	Negative attitude, *N* (%)
Do you think that COVID-19 is a dangerous disease? (Yes)	1,698 (81.5)	385 (18.5)
Do you think that you are vulnerable to COVID-19 infection? (Yes)	1,324 (63.6)	759 (36.4)
Are you interested in knowing the methods of prevention of COVID-19? (Yes)	2,006 (96.3)	77 (3.7)
Do you think that the available information about COVID-19 is sufficient? (No)	995 (47.8)	1,088 (52.2)
Do you think that COVID-19 can be reduced by self-isolation? (Yes)	2,041 (98.0)	42 (2.0)
Do you think that COVID-19 can be reduced by awareness? (Yes)	2,071 (99.4)	12 (0.6)

The majority (89.8%) of the students agreed that COVID-19 had disturbed their daily life. The sociodemographic variables, except for accommodation type, had no significant effect (*P* > 0.05). However, the students living in apartments were significantly more affected by COVID-19 than those living in villas or houses with a yard. More than two-thirds (69.2%) of the students were found to be anxious about being infected with the virus. Age, gender, and college of study significantly affected (*P* ≤ 0.05) the students’ answers to this question. It was found that there was a proportional relationship between age and worry about being infected with COVID-19. In numbers, 73.7%, 69.6%, and 65.5% of the students aged ≥ 25, 20–24.9, and < 20 years showed anxiety about suffering from COVID-19, respectively. Furthermore, female students (70.7%) were more likely to have concern about being infected with COVID-19 than male students (64.4%). Human science students had the greatest worry (73.1%) about being infected by COVID-19, whereas the medical and engineering students (65.0%) had the least (Table 3).

**Table 3:** Students’ life disturbance and fear of being infected by COVID-19 by sociodemographic variables

Demographic variable	Category	*N*	Worried about suffering from COVID-19		Daily life has been disturbed by COVID-19	
			*N* (%)	*P*-value	*N* (%)	*P*-value
Overall	–	2,083	1,441 (69.2)	–	1,871 (89.8)	–
Age (years)	18–19.9	498	326 (65.5)	0.050	445 (89.4)	0.374
	20–24.9	1,304	908 (69.6)		1,167 (89.5)	
	≥ 25	281	207 (73.7)		259 (92.2)	
Gender	Female	1,572	1,112 (70.7)	0.007	1,407 (89.5)	0.399
	Male	511	329 (64.4)		464 (90.8)	
University location	Northern	551	391 (71.0)	0.216	496 (90.0)	0.813
	Middle	1,477	1,017 (68.9)		1,327 (89.8)	
	Southern	55	33 (60.0)		48 (87.3)	
College	Engineering	415	270 (65.1)	0.003	375 (90.4)	0.757
	Medical sciences	535	348 (65.0)		482 (90.1)	
	Agriculture and general sciences	376	270 (71.8)		341 (90.7)	
	Human sciences	757	553 (73.1)		673 (88.9)	
Level of tertiary study	Undergraduate	1,879	1,295 (68.9)	0.436	1,684 (89.6)	0.359
	Postgraduate	204	146 (71.6)		187 (91.7)	
Accommodation type	Villa	106	74 (69.8)	0.893	95 (89.6)	0.001
	Flat	798	545 (68.3)		732 (91.7)	
	House	1,153	803 (69.6)		1,026 (89.0)	
	Others	26	19 (73.1)		18 (69.2)	
Place of residence	City	1,642	1,150 (70.0)	0.059	1,479 (90.1)	0.323
	Village	390	252 (64.6)		344 (88.2)	
	Others	51	39 (76.5)		48 (94.1)	

### Practices of students toward COVID-19.

In general, the students showed low-risk practices toward COVID-19, with a mean score of 84.3%. As with the attitude pattern, two-thirds (67.6%), one-quarter (26.6%), and a small proportion (5.7%) of students showed low-, moderate-, and high-risk practice scores, respectively, toward COVID-19. The practices of students toward COVID-19 were significantly (*P* ≤ 0.05) associated with age, gender, college, level of tertiary study, and accommodation type. In the current study, the age of the students was significantly (*P* ≤ 0.05) associated with safer practices toward COVID-19. Students aged > 25 years had the highest score (86.9%) of behavioral practices toward COVID-19 compared with the younger groups (83.3–84.1%). It was notable that more than three-quarters (75.8%) of the students aged ≥ 25 years showed low-risk practices toward COVID-19 than students aged > 20 (63.9%) years or those aged 20–24.9 (67.3%) years.

Female students showed a significantly higher low-risk practice score (85.0%) than male students (82.2%) toward COVID-19. In addition, 69.9% of females showed low-risk practices toward COVID-19 compared with 60.7% of male students. The students of medical science schools (85.6%) and agricultural and applied sciences schools (85.2%) scored the highest on practices toward COVID-19 compared with human science (83.3%) and engineering (83.9%) students. In addition, 71.0% of medical science students and 71.8% of agricultural and applied sciences students showed low-risk practices toward COVID-19 compared with 63.1% of engineering students and 65.7% of human science students.

The average of low-risk practice score of postgraduate students (86.9%) toward COVID-19 was higher than that of undergraduate students (84.0%). More than three-quarters (76.5%) of the postgraduate students showed low-risk practices than two-thirds of undergraduate students (66.7%). Approximately 72.6% of the students living in villas showed low-risk practices toward COVID-19 with an average score of 86.0%, compared with 68.3% of the students living in houses with an average score of 84.2% and 66.5% of students living in apartments with an average score of 84.4% (Table 4).

**Table 4:** Behavioral practice categories and scores of university students in Jordan toward COVID-19

Demographic variable	Category	*N*	Practice category, *N* (%)			Practice mean (%)	SD	χ^2^	*P*-value
			High-risk	Moderate-risk	Low-risk				
Overall	–	2,083	119 (5.7)	555 (26.6)	1,409 (67.6)	84.3	11.7	–	–
Age (years)	18–19.9	498	38 (7.6)	142 (28.5)	318 (63.9)	83.3	12.3	14.111	0.007
	20–24.9	1,304	71 (5.4)	355 (27.2)	878 (67.3)	84.1	11.6		
	≥ 25	281	10 (3.6)	58 (20.6)	213 (75.8)	86.9	10.6		
Gender	Female	1,572	72 (4.6)	401 (25.5)	1,099 (69.9)	85.0	10.8	22.366	0.000
	Male	511	47 (9.2)	154 (30.1)	310 (60.7)	82.2	13.9		
University location	Northern	551	22 (4.0)	136 (24.7)	393 (71.3)	85.5	10.9	7.542	0.110
	Middle	1,477	94 (6.4)	407 (27.6)	976 (66.1)	83.9	12.0		
	Southern	55	3 (5.5)	12 (21.8)	40 (72.7)	84.1	11.2		
College	Engineering	415	18 (4.3)	135 (32.5)	262 (63.1)	83.9	11.5	18.797	0.005
	Medical sciences	535	27 (5.0)	128 (23.9)	380 (71.0)	85.2	10.9		
	Agriculture and general sciences	376	17 (4.5)	89 (23.7)	270 (71.8)	85.6	10.5		
	Human sciences	757	57 (7.5)	203 (26.8)	497 (65.7)	83.3	12.7		
Level of tertiary study	Undergraduate	1,879	112 (6.0)	514 (27.4)	1,253 (66.7)	84.0	11.9	8.303	0.016
	Postgraduate	204	7 (3.4)	41 (20.1)	156 (76.5)	86.9	9.7		
Accommodation type	Villa	106	4 (3.8)	25 (23.6)	77 (72.6)	86.0	10.0	12.779	0.047
	Flat	798	42 (5.3)	225 (28.2)	531 (66.5)	84.4	11.4		
	House	1,153	68 (5.9)	297 (25.8)	788 (68.3)	84.2	11.9		
	Others	26	5 (19.2)	8 (30.8)	13 (50.0)	77.5	17.5		
Place of residence	City	1,642	88 (5.4)	428 (26.1)	1,126 (68.6)	84.6	11.2	6.183	0.186
	Village	390	25 (6.4)	112 (28.7)	253 (64.9)	83.4	13.2		
	Others	51	6 (11.8)	15 (29.4)	30 (58.8)	82.2	13.6		

Among 20 questions about practices toward COVID-19, the majority (81.9–98.8%) of students correctly answered 16 questions. These practices included covering the nose with tissue when coughing or sneezing followed by throwing away the used tissue, avoidance of contact with infected persons or other people when they have flu-like symptoms, avoidance of crowded places and hand-shaking, practicing physical-distancing, seeking medical advice when having symptoms of COVID-19, and handwashing with water and soap in different situations. However, about two-thirds (66.8%) of students washed their hands for ≥ 20 seconds, and exactly 64.9% of the students washed their hands before touching their eyes or nose. Conversely, only 39.8% of the students used face masks to protect themselves from COVID-19 (Table 5).

**Table 5:** Responses about practices toward COVID-19 among university students in Jordan

Question (low-risk practice)	Low-risk practice, *N* (%)	High-risk practice, *N* (%)
Do you cover your nose with tissue when coughing and sneezing? (Yes)	1,975 (94.8)	108 (5.2)
Do you throw away the used tissue into the bin directly after use? (Yes)	1,931 (92.7)	152 (7.3)
When do you wash your hands to protect yourself from COVID-19? After toilet (Yes)	1,995 (95.8)	88 (4.2)
After touching the personal items of someone who has a cough and/or cold (Yes)	1,861 (89.3)	222 (10.7)
Before touching eyes or nose (Yes)	1,352 (64.9)	731 (35.1)
After shaking hands with others (Yes)	1,706 (81.9)	377 (18.1)
After touching common contact surfaces such as doorknobs or elevator buttons (Yes)	1,733 (83.2)	350 (16.8)
Before eating (Yes)	1,882 (90.4)	201 (9.6)
When you come back home (Yes)	1,970 (94.6)	113 (5.4)
Before sleeping (No)	830 (39.8)	1,253 (60.2)
What do you use to wash your hands to protect yourself from COVID-19? (water and soap)	2,052 (98.5)	31 (1.5)
How much time you spend in washing your hands? (≥ 20 seconds)	1,391 (66.8)	692 (33.2)
Do you use disinfectant or hand gel to wash your hands to protect yourself from COVID-19? (Yes)	1,844 (88.5)	239 (11.5)
Do you use face mask when leaving home? (Yes)	829 (39.8)	1,254 (60.2)
Do you avoid crowded places to protect yourself from COVID-19? (Yes)	2,014 (96.7)	69 (3.3)
Do you practice social distancing to protect yourself from COVID-19? (Yes)	1,890 (90.7)	193 (9.3)
Do you avoid contact with a positively confirmed COVID-19 patient? (Yes)	2,059 (98.8)	24 (1.2)
Do you avoid hand-shaking to protect yourself from COVID-19? (Yes)	1,861 (89.3)	222 (10.7)
If you have flu symptoms, do you avoid contact with other people? (Yes)	1,932 (92.8)	151 (7.2)
Would you seek medical advice if you experience any symptoms of COVID-19? (Yes)	2,016 (96.8)	67 (3.2)

## DISCUSSION

Governments, health authorities, and scientists worldwide are trying to manage and contain COVID-19 infections. It is evident that community awareness regarding preventive measures against COVID-19 plays a crucial role in reducing disease transmission. It seems that the affirmation of COVID-19 as a pandemic infection by the WHO^6^ had a substantial impact on the attitudes and practices of the students in the current study, regardless of different personal sociodemographic characteristics. The participating students showed good knowledge about COVID-19 with an average score of 80.7%,^17^ which may have enhanced their perspectives and behavioral practices concerning COVID-19 in the current study. Most students showed a positive attitude and low-risk practices toward COVID-19, with overall scores of 81.1% and 84.3%, respectively. Approximately two-thirds of the students showed positive scores in the attitude and low-risk practices sections of the survey toward COVID-19. Alzoubi et al.^18^ found a similar attitude with an overall score of 82% among undergraduate students at the University of Mutah in Jordan, but they found a notably lower practice score of 78% toward COVID-19. This difference in behavioral practices could be attributed to the difference in the time the studies were conducted. The current study was completed a few days after the government had reported several COVID-19 cases in Jordan and the infection appeared to be a serious issue with high risk. It is generally accepted that undergraduate students are younger and less knowledgeable than postgraduate students, and thus they are less likely to follow conscientious low-risk behavioral practices. It has been observed that acceptable risk varies according to gender and age, with men and younger people being more likely to engage in higher risk practices.^19^ It is worth mentioning that the percentage of females who participated in the current study was quite large and may explain why the behavioral low-risk practice score in this study is higher than that reported by Alzoubi et al.^18^ Zhong et al.^19^ also found a significant association among males, young people, and the use of unsafe practices toward COVID-19. It was also found that there was a significant association between students who were worried they might be infected with COVID-19 and the use of low-risk practices. Female and older students were more anxious about being infected with COVID-19 than other students, and this may explain why these groups exhibited better behavioral practices.

In another study, Huynh et al.^20^ reported that 93.3% of healthcare workers in Vietnam had acceptable attitudes toward COVID-19. Several factors including the disease fatality rate, its virulence and health complications that can arise, and its highly contagious nature affect the attitudes and practices of people everywhere. The current study was conducted after the WHO announced that COVID-19 had become a pandemic.^6^ However, the infection was still transmitting slowly in Jordan.^7^ The attitudes and practices of the respondents revealed that it was believed COVID-19 could be prevented by different measures such as frequently handwashing with water and soap, following good respiratory hygiene and cough etiquette, avoidance of contact with infected persons or other people with flu-like symptoms, avoidance of hand-shaking and crowded places, practicing physical-distancing, and seeking medical advice if symptoms of COVID-19 develop. These measures are well recognized to be cornerstones for the prevention of transmission of infectious respiratory diseases such as COVID-19. It is likely that in addition to government regulations, positive attitudes and low-risk practices among students, as a major part of Jordan’s population, could play a significant role in reducing COVID-19 transmission in Jordan where only 987 cases and nine deaths had been reported at the time of writing this article.^7^

Although the medical students had a significantly lower level of fear (65.0%) about being infected with COVID-19, they displayed a higher proportion of low-risk practices along with the students of agriculture and general sciences. This could be attributed to the fact that the medical students had more access to accurate knowledge, and they were more familiar with low-risk practices than other students. It was found that medical students had significantly greater knowledge about COVID-19 than students from other schools of study.^17^ By contrast, Alzoubi et al.^18^ found that there was no significant difference between the practices of medical and nonmedical students. The vast majority (89.8%) of the students agreed that their daily life had been disturbed by COVID-19. This was likely because of the complete lockdown of Jordan, through the imposition of the state of emergency and a strict curfew law.

Unfortunately, in the present study, discrepancies were found in some attitudes and practices of university students toward COVID-19. About 6.5% and 5.7% of the students displayed negative attitudes and high-risk practices, respectively, toward COVID-19. More than one-third (36.4%) of the students incorrectly believed that they were not susceptible to COVID-19 and half (18.5%) of those believed that COVID-19 was not a perilous disease. In addition, a significant proportion (35.1%) of the students were not washing their hands before touching their eyes or nose, after shaking hands with others (18.1%), or after touching common contact surfaces such as doorknobs or elevator buttons (16.8%). These practices could play a significant role in the transmission of COVID-19 because they were confirmed as factors contributing to its transmission.^6,11^

Surprisingly, one-third (33.2%) of the students reported that they are practicing inappropriate handwashing techniques because of use of a contact time of less than 20 seconds which is the minimum recommended by the CDC.^13^ The WHO declared that a suitable duration for the entire handwashing procedure using water and soap should be 40–60 seconds.^6^ In the current study, only a small proportion (4.4%) of the students chose a minimal time of 40 seconds for handwashing. Furthermore, a significant percentage (60.2%) of students did not wear face masks when going out. This may have resulted from the inconsistent recommendations regarding the use of face masks by the general public in community settings by different health authorities including the WHO, CDC, and ECDC.^21^ According to the WHO recommendation, healthy people should wear face masks when they are taking care of a COVID-19–infected person.^6^ The CDC at this point in time did not recommend that healthy people should use face masks.^15^ On the other hand, the ECDC stated that using face masks in public could be considered, mainly in crowded, enclosed spaces.^16^ Face masks may reduce the transmission of COVID-19 infection by decreasing droplet spread from infected persons or from asymptomatic, infected people before the onset of symptoms.^16,21^

Furthermore, about half (47.8%) of the students correctly recognized that the available data about COVID-19 are insufficient. Therefore, it is necessary that the Ministry of Health in Jordan and other health planning groups comprehensively review and update the available data and build appropriate COVID-19 health educational programs taking into account the knowledge, attitudes, and practices that already exist within the community, including university students, healthcare workers, and other sectors of the population to promote safer lifestyles and prevent transmission of COVID-19 and other infectious diseases.

In conclusion, most of the students from different universities in Jordan exhibited positive attitudes and low-risk practices regarding COVID-19, with average scores of 81.1% and 84.3%, respectively. However, a small proportion of students showed negative attitudes or high-risk practices. Sociodemographic variables including age, gender, college of study, level of tertiary study, and accommodation type were significantly associated with the students’ practices but not with their attitudes regarding COVID-19. Female, older, medical, or postgraduate students showed significantly higher practice scores toward COVID-19 than other groups. Furthermore, most of the students confirmed that COVID-19 had interrupted their daily life, and about two-thirds of the students were worried they might become infected with COVID-19. The results of this study could serve as a foundation for the development of counseling and health training programs to enhance the engagement of university students in controlling the spread of infectious diseases including COVID-19.
